# Characteristics and outcomes of women initiating ART during pregnancy versus breastfeeding in Option B+ in Malawi

**DOI:** 10.1186/s12889-016-3380-7

**Published:** 2016-08-04

**Authors:** M. Landes, S. Sodhi, A. Matengeni, C. Meaney, M. van Lettow, A. K. Chan, J. J. van Oosterhout

**Affiliations:** 1 Dignitas International, Zomba, Malawi; 2 Department of Family and Community Medicine, University of Toronto, Toronto, Canada; 3 Dalla Lana School of Public Health, University of Toronto, Toronto, Canada; 4 Division of Infectious Diseases, Department of Medicine, Sunnybrook Health Sciences Centre, University of Toronto, Toronto, Canada; 5 Department of Medicine, University of Malawi College of Medicine, Blantyre, Malawi

**Keywords:** Option B+, Prevention of mother to child transmission of HIV, ART initiation, Malawi, Pregnancy, Breastfeeding, HIV

## Abstract

**Background:**

Malawi adopted the PMTCT strategy ‘Option B+’ in 2011, providing life-long ART for all HIV-infected pregnant and breastfeeding women. We explored differences in characteristics and outcomes of women initiating ART during pregnancy versus breastfeeding.

**Methods:**

We conducted a retrospective cohort analysis of women in Zomba District, southern Malawi, from January 2012- September 2013. Data were extracted from the Zomba District Observational Cohort Study, a surveillance project collecting data from standardized Ministry of Health ART monitoring tools.

**Results:**

1986 (67.2 %) women initiated ART during pregnancy and 969 (32.8 %) during breastfeeding. Women initiating ART in breastfeeding were more likely to be > 30 years (aOR = 1.33, 95 % CI1.11–1.59, *p =* 0.003) and have WHO Stage 3/4 (aOR = 2.74, 95 % CI1.94–3.87, *p <* 0.001*).*

Eighteen (0.6 %) deaths occurred and 942 (31.9 %) women defaulted ART. ‘Early’ death (< 30 days) occurred in 3 (0.1 %) women and 449 (16.4 %) women defaulted early. Death/default < 30 days was more likely among women initiating ART during pregnancy (aOR 1.62, 95 % CI1.28–2.05, *p <* 0.001) or < 30 years old (aOR 1.27, 95 % CI 1.02–1.57, *p =* 0.03) and was less likely among those with WHO Stage 3/4 (aOR 0.30, 95 % CI 0.15–0.60, *p <* 0.001).

Using Kaplan-Meier estimators to investigate time to death/default, we showed a sharp drop in death/default-free survival probability at time zero, yet survival probability decreased in a nearly linear manner after this initial period of high default. Women under 30 years had increased rates of death/default over time (log rank test: *p <* 0.001), however no significant differences were observed in death/default over time associated with timing of ART initiation, documented clinical stage at initiation, health clinic size or adherence rates.

**Conclusions:**

Many women in Malawi started ART during breastfeeding within Option B+ and were older and had more advanced WHO Clinical Staging. This represents a missed PMTCT opportunity to initiate treatment early in pregnancy. Early defaulting is identified as a challenge within Option B+, and was more likely among younger women and those initiating ART in pregnancy. Targeted research to understand factors associated with uptake of ART during pregnancy and retention in care could improve the efficacy of Option B+ in Malawi.

## Background

In 2011, the Malawi Ministry of Health (MoH) adopted a national program for the prevention of mother to child transmission (PMTCT) of HIV called ‘Option B+’, which provides life-long antiretroviral therapy (ART) for all HIV-infected pregnant and breastfeeding women regardless of clinical or immunological stage.

Since the introduction of Option B+, aggregated national data have shown a 6-fold increase in the number of pregnant women initiating ART in Malawi with an estimated 28,000 women accessing ART annually across the country [1].

Additionally, national program monitoring suggests a fairly high level of retention among women initiating ART in Option B+, with estimated retention rates of 72 % at 12 months and 68 % at 24 months [1]. Recent studies however highlight challenges around early defaulting in women initiating ART through Option B+ with women either failing to start ART (i.e. immediate defaulting) or defaulting at less than 6 months [2, 3].

While national guidelines encourage early identification of HIV-infected women in pregnancy through testing and early initiation of ART at antenatal clinics (ANC), program  data consistently show that at least one-fifth of women initiate ART later in the breastfeeding period [1]. This delayed initiation of ART represents a missed opportunity for preventing HIV transmission in utero, during delivery and in the early postpartum stages. Reasons for delayed ART initiation among these women may include late or missed identification at ANC and delivery (including non-attendance at ANC or refusal of HIV testing), incident HIV infection in pregnancy or women re-initiating ART after defaulting during pregnancy [1].

Limited description exists of the differences between women initiating ART during pregnancy or breastfeeding, both in terms of characteristics associated with the timing of ART initiation and their outcomes within the Option B+ program. We conducted a retrospective cohort analysis within the Zomba Observational Cohort Study, a population-based observational cohort of ART patients in southeastern Malawi, in order to investigate differences between the characteristics of women either initiating ART in pregnancy or during the breastfeeding period. Additionally we investigated the relationship between timing of ART initiation and time to death or default.

## Methods

### Study design and setting

A retrospective cohort analysis was conducted including all women in the Zomba District of southern Malawi who initiated ART from January 1, 2012 through September 30, 2013. Malawi is a developing country in sub-Saharan Africa with an HIV prevalence of 10.6 % among 15–49 year olds. By the end of 2014, more than 500,000 persons were alive on ART, indicating that close to 50 % of all HIV-infected Malawians were on ART [1]. PMTCT care was provided in all health centres according to the Option B+ strategy as per national MoH guidelines starting in 2011 [4]. Pregnant or breastfeeding mothers received HIV-testing and counselling in antenatal care clinics or Under-5 Clinics and HIV-infected women were offered life-long ART regardless of clinical or immunological stage. All women were initiated and followed either in the ANC clinic or in a linked ART clinic.

### Data collection

Demographic, clinical and outcome data were extracted from the Zomba District Observational Cohort Study database. This cohort study routinely collects individual level HIV care information from 31 health centres (8 urban, 23 rural) in Zomba District via standard Malawi MoH ART monitoring tools. Methods for data collection have been described previously [5]. Ethics approval for the study was received from the Malawi National Health Services Research Committee, Malawi and the University Health Network, Toronto, Canada.

Patient characteristics were categorized as: age (< 30 years; > = 30 years), size of health facility at which they received care (<100 patients on ART; 100–500 patients on ART; 500+ patients on ART), WHO clinical stage on ART initiation (Stage 1; Stage 2; Stage 3–4; unknown stage) and ART adherence rates defined as percentage of self-reported pills remaining out of those prescribed from the last visit (< 95 % adherence; > = 95 % adherence). Patient outcomes were classified as either: alive and on ART, dead or defaulted (failed to visit their clinic within 2 months after the next appointment date or stopped ART).

### Statistical methods

Descriptive statistics were used to summarize characteristics of our sample. Contingency tables and Pearson’s chi-squared tests were used to describe unadjusted relationships between timing of ART initiation and covariates. Multivariate logistic regression was used to estimate unadjusted/adjusted odds of initiating ART during breastfeeding.

Further, bivariate and adjusted multivariate analysis were used to investigate timing of ART initiation and its impact on time to death/default (as a composite outcome variable). We considered two operational definitions of death/default: first, we investigated whether a women had died/defaulted within 30 days of ART initiation, censoring those women with no observed outcome (lacking confirmed data) within 30 days. Similar methods for categorical data analysis as those described above were used to investigate the association between covariates and the odds of death/default within 30 days of ART initiation. Sensitivity analyses of death/default within 90 days and 365 days of ART initiation, were performed and inferences from models fit to these outcome data were similar to those from the 30 day response models. As such, results of the sensitivity analyses are not shown. Additionally, we explored the association between death/default-free survival using Kaplan-Meier survival analysis estimators and log-rank tests. These methods are capable of accounting for censoring of the outcome variable (death/default) and complement the categorical data analysis approach outlined above. All statistical analyses were conducted in SAS version 9.4 (SAS Corporation, Cary, North Carolina).

## Results

### Description of cohort

Overall, 2955 women initiated ART during the study period. Data were recorded for initial ARV regimen in 2730 (92.4 %) women, of which 2658 (97.4 %) started the Option B+ first line ART regimen (a generic, one-tablet formulation of tenofovir, lamivudine and efavirenz). The median age in our sample was 29 years (IQR: 24–33). WHO Clinical Stage at enrolment was recorded for 2153 (72.9 %) women: 1777 (82.5 %) of whom were WHO Clinical Stage 1 and 1983 (92.1 %) were either Stage 1 or Stage 2. Adherence data were available for 2138 (72.4 %), women of whom 2090 (97.8 %) had an adherence of > =95 %.

### Initiation of ART

Of the 2955 women in the sample, 1986 (67.2 %) initiated ART during pregnancy and 969 (32.8 %) during breastfeeding (Table 1). Women initiating ART in breastfeeding were more likely to be older (> 30 years; aOR = 1.33, 95 % CI 1.11, 1.59, *p =* 0.003) and have advanced WHO Clinical Stage (Stage 2 or Stage 3 and 4) at the time of enrolment (aOR = 1.59, 95 % CI 1.15–2.18, *p =* 0.005 and aOR = 2.74, 95 % CI 1.94–3.87, *p <* 0.001*)* (Table 2).

**Table 1:** Maternal characteristics by timing of ART initiation in Option B+ Programme

	ART Initiation During Pregnancy *N =* 1986	ART Initiation During Breastfeeding *N =* 969	*P*-value
Age			
< 30 years	1293 (65.4 %)	565 (58.5 %)	< 0.001
> 30 years	685 (34.6 %)	401 (41.5 %)	
Health Facility Size^a^			
Small (< 100)	45 (2.3 %)	29 (3.0 %)	0.06
Medium (100–500)	1674 (84.3 %)	836 (86.3 %)	
Large (> 500)	267 (13.4 %)	104 (10.7 %)	
WHO Clinical Stage at Initiation			
Stage 1	1241 (62.5 %)	536 (55.3 %)	< 0.001
Stage 2	119 (6.0 %)	87 (9.0 %)	
Stage 3–4	75 (3.8 %)	95 (9.8 %)	
Not documented	551 (27.7 %)	251 (25.9 %)	
Adherence Rates			
> 95 %	1330 (97.4 %)	766 (98.3 %)	0.18
< 95 %	35 (2.6 %)	13 (1.7 %)	

^a^Based on number of patients on ART at health facility

**Table 2:** Association between maternal characteristics and the probability of initiating ART during breastfeeding

	Unadjusted Odds Ratios			Adjusted Odds Ratios		
	OR	95 % CI	*P*-value	aOR	95 % CI	*P*-value
Age						
< 30 years	---	---	---	---	---	---
> 30 years	1.34	1.14, 1.57	< 0.001	1.33	1.11, 1.59	0.003
Health Facility Size^a^						
Small (< 100)	---	---	---	---	---	---
Medium (100–500)	0.78	0.48, 1.25	0.29	0.78	0.45, 1.35	0.37
Large (> 500)	0.60	0.36, 1.02	0.06	0.65	0.36, 1.17	0.15
WHO Clinical Stage at Initiation						
Stage 1	---	---	---	---	---	---
Stage 2	1.69	1.26, 2.27	< 0.001	1.59	1.15, 2.18	0.005
Stage 3–4	2.93	2.13, 4.03	< 0.001	2.74	1.94, 3.87	< 0.001
Not documented	1.06	0.88, 1.26	0.56	1.18	0.95, 1.47	0.14
Adherence Rates						
= > 95 %	---	---	---	---	---	---
< 95 %	1.55	0.82, 2.95	0.18	1.50	0.78, 2.88	0.22

^a^ Based on number of patients on ART at health facility

### Death or treatment default

Among the 2955 women in the cohort, we observed 18 (0.6 %) confirmed deaths of which 13 (72.2 %) occurred among women in WHO Clinical Stage 1 or 2 at the time of ART initiation. 1995 (67.5 %) women were observed as retained on ART and 942 (31.9 %) women were recorded as having defaulted ART, of whom 396 (42.0 %) did not return after the ART initiation visit.

In the following section we describe early death/default occurring within 30 days of ART initiation. We observed early death in 3 (0.1 %) women and early defaulting in 449 (16.4 %) women. Table 3 shows bivariate and multivariate associations between maternal characteristics and the probability of death or default within 30 days. Death or default by 30 days was more likely among women initiating ART during pregnancy (aOR 1.62, 95 % CI 1.28–2.05, *p <* 0.001) and who were less than 30 years old (aOR 1.27, 95 % CI 1.02–1.57, *p =* 0.03). Women with WHO Stage 2 and 3–4 had lower adjusted odds of 30 day death or default compared to women with WHO Stage 1 (aOR 0.62, 95 % CI 0.39–0.98, *p =* 0.04; aOR 0.30, 95 % CI 0.15–0.60, *p <* 0.001 respectively).

**Table 3:** Factors associated with death/default at 30 days (*N =* 2670)

	Unadjusted Odds Ratios			Adjusted Odds Ratios		
	OR	95 % CI	*P*-value	aOR	95 % CI	*P*-value
Timing of ART Initiation						
In Pregnancy	1.72	1.37, 2.17	< 0.001	1.62	1.28, 2.05	< 0.001
In Breastfeeding	---					
Age						
< 30 years	1.33	1.07, 1.65	0.01	1.27	1.02, 1.57	0.03
> 30 years	---					
Health Facility Size						
Small (< 100)	---	---	---			
Medium (100–500)	1.00	0.52, 1.92	0.99			
Large (> 500)	1.19	0.59, 2.41	0.62			
WHO Clinical Stage at Initiation						
Stage 1	---	---	---	---	---	---
Stage 2	0.58	0.37, 0.92	0.02	0.62	0.39, 0.98	0.04
Stage 3–4 Not documented	0.26 1.15	0.13, 0.52 0.91, 1.47	< 0.001 0.24	0.30 1.18	0.15, 0.60 0.93, 1.50	< 0.001 0.18

Adjusted for timing, age and clinical stage

If a woman was not observed for at least 30 days, they are removed from the analysis

**Fig. 1:**
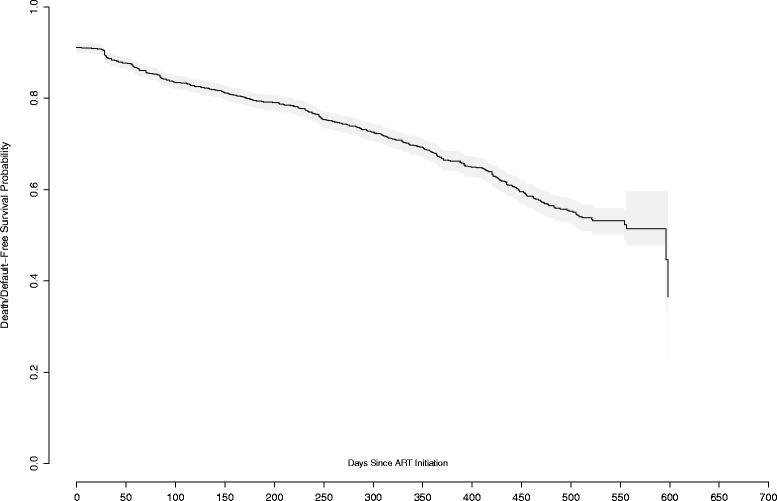
Kaplan Meier estimates of death/default free survival probability in the overall cohort

In Fig. 1, we further investigate death/default free survival experiences of women in this cohort using Kaplan-Meier estimators. A large number of women had zero follow-up time which results in a sharp drop in the death/default free survival probability at time zero. The estimated survival probability decreases in a nearly linear manner after the initial period of high default. Overall, the median death/default free survival time in this cohort is estimated to be 596 days.

**Fig. 2:**
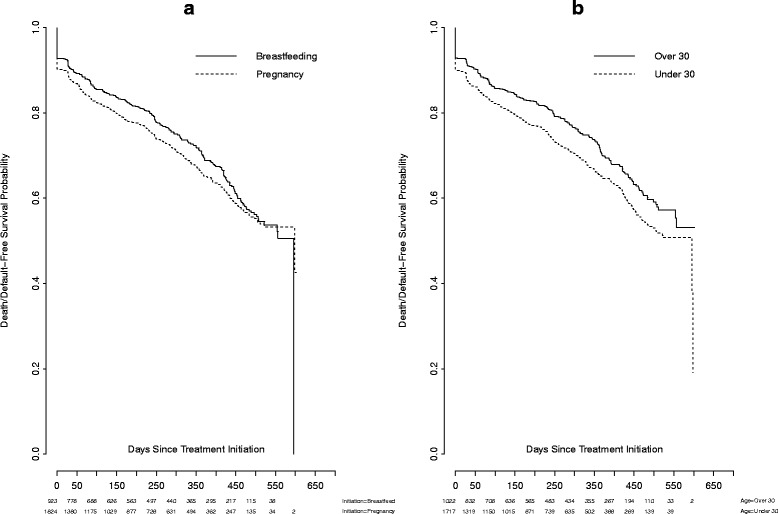
Kaplan Meier estimates of death/default free survival probability stratified by **a** timing of treatment initiation (breastfeeding versus pregnancy) and **b** age (less than 30 years of age versus greater

Figure 2a-b illustrate the association between maternal characteristics and death/default free survival. In Fig. 2a, we observed that the protective effect from death/default of initiating ART during breastfeeding was attenuated after 1-year following treatment initiation and we see that at 1-½ years post ART initiation the probability of death/default was roughly the same in women initiating during pregnancy/breastfeeding. Ultimately, we fail to reject the null hypothesis that death/default-free survival between women initiating ART pregnancy and breastfeeding is different over the duration of follow up time in the study (log-rank test: *p =* 0.12). However, Fig. 2b suggests that women under 30 years of age have reduced death/default free survival compared to older women (log rank test: *p <* 0.001). This finding is consistent across the time course of the study. We observed no significant differences in the death/default-free survival of women in documented different clinical stages, from different sized health facilities, or of women with different treatment adherence rates (Kaplan-Meier analysis results not shown).

## Discussion

In this study, we report on a large number of HIV-infected pregnant or breastfeeding women initiating ART under the Option B+ strategy at a time when it was well established in the rural southeast health-zone of Malawi. We found that a high percentage of women (32.8 %) initiated ART during breastfeeding and that this was associated with older age and more advanced HIV disease (WHO Stage > 1). Overall recorded mortality was very low (0.6 %), but overall defaulting was considerable (31.9 %). We also found that risk of early death/defaulting (< 30 days) was higher in women who started ART in pregnancy and in younger women (< 30 years) and lower in those with more advanced HIV disease (WHO Stage > 1). However, while initial differences are seen in early defaulting between women starting ART during pregnancy and breastfeeding, survival analysis demonstrated that this difference faded at longer follow up and was ultimately non-significant.

By using routinely collected data from the Zomba Observational Cohort Study, we are able to describe individual characteristics and outcomes for this cohort. Our data reflect national monitoring reports in that while the majority of women initiated ART during pregnancy, an important proportion continue to have delayed initiation of ART during breastfeeding [1]. We report that women initiating ART during breastfeeding were more likely to be older and to have more advanced HIV disease. This suggests there may be differing motives for entering care among these two groups. It is possible that women initiate ART during breastfeeding period because of symptoms that bring them to seek care at a health facility whereas routine HIV-testing in pregnancy captures a larger proportion of asymptomatic women. Regardless, the delayed initiation of ART within the PMTCT cascade and increased prevalence of advanced HIV disease (suggesting higher HIV1-RNA levels) among these women raises concerns for increased mother to child transmission of HIV. Interventions to encourage earlier presentation to ANC for HIV testing and ART initiation may improve outcomes in Option B+.

Overall, we report moderately high rates of retention and high levels of self-reported adherence among a largely asymptomatic, and predominantly rural cohort. These results are comparable to facility level nation-wide reports from the Malawi HIV programme and to a study that utilized individual level analyses from mostly urban, large Malawian health facilities with electronic monitoring systems and one further study from urban Lilongwe [1–3, 6]. Similar to the reports mentioned above we also show that the largest risk for defaulting exists early after starting ART [1–3].

Additionally, we report that women initiating ART in pregnancy were more likely to be early defaulters than women initiating ART during breastfeeding. This agrees with a recent study from Malawi in which women initiating ART during pregnancy had higher default rates at 6 months than those initiating during breastfeeding [2]. It is possible that timing of HIV diagnosis and ART initiation itself may be a factor associated with risk of early defaulting among women in Option B+. Recent evidence describes that both women and health care providers identify that a new HIV diagnosis during routine antenatal screening can be accompanied by varying degrees of shock and denial and may lead to difficulty accepting immediate initiation of lifelong treatment, in particular among the mostly asymptomatic pregnant women at ANC clinics [7, 8]. Furthermore, it has recently been reported that receiving a new HIV diagnosis and ART initiation on the same day increases this risk of early defaulting [2, 9] and that the model of care for PMTCT at health centres in Malawi, including how timing of HIV testing and ART initiation is structured, can impact overall uptake and retention [10].

Given that we report that women who initiate ART during breastfeeding were more likely to be older and present with more advanced HIV disease, lower rates of early defaulting in this group may also reflect differing individual patient motivations for initiating and continuing on ART. Tweya et al. showed similar lower rates of loss to follow-up among women initiating during breastfeeding in Malawi [11]. While illness has been cited as a reason for defaulting ART in Option B+ in Malawi [11], a further recent qualitative study in Malawi notes that both fear of future sickness or a current decline in health prompted women who had already stopped ART in Option B+ to re-start later (either during pregnancy or breastfeeding) [12]. Further research to understand patient characteristics, experience and motivations that lead to improved earlier uptake of ART and subsequent adherence should be incorporated into programmatic adaptations of Option B+.

Finally, we highlight that younger age remained the most consistent risk factor for defaulting both early after initiation and over the course of follow-up in the study. In the previously mentioned study by Tweya et al., younger age (< 24 years) was similarly associated with an increased loss to follow-up among an urban Option B+ cohort in Malawi [11]. Reasons for higher defaulting among younger women remain to be understood in women in Option B+ and further research could identify both reasons for this association along with potential mechanisms for targeting support for younger women in care.

### Limitations

There are several limitations to this study. Lack of a defaulter tracing system may underestimate mortality for women initiating within Option B+ as well as their retention, as recent Malawian data found that a relevant proportion of traced defaulters had either died or self-transferred to other care [10]. Additionally, we relied on routine data collection using national standardized ART monitoring forms, which may have limited the quality of the data. Specifically, as women are enrolled in Option B+ regardless of stage, data on WHO clinical staging may be less reliably documented and was missing in just over 1/4 of all women. Finally, adherence measures on standardized ART cards are limited to self-report of doses missed since last appointment.

## Conclusions/recommendations

While early identification and initiation of ART among pregnant women is encouraged in the Option B+ Program in Malawi, many women continue to start ART during breastfeeding. These women were older, more often in WHO Clinical Stage 2, 3 or 4 and had a lower risk of early death/default compared to those starting during pregnancy. Starting ART during breast feeding represents a missed PMTCT opportunity during the antenatal phase and delivery. Additionally, early defaulting is identified as a challenge within Option B+, and was more likely among younger women and those initiating ART in pregnancy. Targeted research to understand factors associated with the uptake of ART during pregnancy and retention in care could improve the efficacy of Option B+ in Malawi by informing targeted programmatic interventions.

## References

[1] Malawi Ministry of Health (2014). Integrated HIV Program Report: October 2014-December 2014.

[2] Tenthani L (2014). Haas Ad, Tweya H, et al. Retention in care under universal Antiretroviral Therapy for HIV Infected Pregnant and Breastfeeding Women (“Option B+”) in Malawi. AIDS.

[3] Haas AD, Tenthani L, Msukwa M (2015). Retention on lifelong ART under Option B+ in Malawi, Oral presentation, IAS 2015.

[4] Malawi Ministry of Health (2014). Clinical Management of HIV in Children and Adults.

[5] Negin J, van Lettow M, Semba M (2011). Anti-Retroviral Treatment Outcomes among Older Adults in Zomba District, Malawi. PLoS One.

[6] Kim MH, Ahmed S, Hosseinipour MC (2015). Implementation and operational research: the impact of option B+ on the antenatal PMTCT cascade in Lilongwe, Malawi.. J Acquir Immune Defic Syndr.

[7] Stinson K, Myer L (2012). Barriers to initiating antiretroviral therapy during pregnancy: a qualitative study of women attending services in Cape Town, South Africa. African J AIDS Res.

[8] Cataldo F (2013). Exploring women and health care workers experiences in the context of PMTCT Option B Plus in Malawi, Oral presentation.

[9] Chan AK, Kanike E, Bedell R (2016). Same day HIV diagnosis and antiretroviral therapy initiation affects retention in Option B+ prevention of mother-to-child transmission services at antenatal care in Zomba District, Malawi. J Int AIDS Soc.

[10] Van Lettow M, Bedell R, Mayuni I (2014). Towards elimination of mother-to-child transmission of HIV: performance of different models of care for initiating lifelong antiretroviral therapy for pregnant women in Malawi (Option B+). J Int AIDS Soc.

[11] Tweya H, Gugsa S, Hosseinipour M (2014). Understanding factors, outcomes and reasons for loss to follow-up among women in Option B+ PMTCT programme in Lilongwe, Malawi. Trop Med Int Health.

[12] Kim M, Zhou A, Mazenga A (2015). Why Did I Stop? Barriers and Facilitators to Uptake and Retention in Option B+ HIV Care in Lilongwe, Malawi. Poster presentation, International AIDS Society Conference.

